# Four new species of *Microdochium* (*Microdochiaceae*, *Xylariales*) based on morphology and multilocus phylogeny from Hainan, China

**DOI:** 10.3897/mycokeys.133.190511

**Published:** 2026-06-02

**Authors:** Yu-Jiao Wang, Xi-Gang Yan, Shi Wang, Yang Jiang, Yu-Xin Shang, Wen-Wen Liu, Xing-Sheng Wang, Jin-Yan Sheng, Xiu-Guo Zhang, Cong-Cong Ai

**Affiliations:** 1 College of Life Sciences, Shandong Normal University, Jinan, 250358, China College of Life Sciences, Shandong Normal University Jinan China; 2 Shandong Provincial Key Laboratory for Biology of Vegetable Diseases and Insect Pests, College of Plant Protection, Shandong Agricultural University, Taian, 271018, China College of Plant Protection, Shandong Agricultural University Taian China

**Keywords:** *
Ascomycetes
*, fungal diversity, new taxa, phylogenetic analysis, taxonomy

## Abstract

*Microdochium* fungi are globally distributed and function as plant pathogens, saprobes, or endophytes, infecting a wide variety of plant hosts. In the present study, four novel *Microdochium* species collected from diseased leaves of *Bambusoideae* sp., *Acronychia pedunculata*, and *Symplocos sumuntia* in Hainan Province, China, were isolated, identified, and formally described. Species delimitation was achieved through a combination of molecular phylogenetic inference and morphological comparison with closely related taxa. Phylogenetic analyses were conducted on concatenated ITS, LSU, RPB2, and TUB2 sequence datasets using both maximum likelihood (ML) and Bayesian inference (BI) methods. The results demonstrate that these four species form well-supported, independent monophyletic lineages that are clearly distinct from all previously described *Microdochium* species. Morphological examination of colony characteristics, conidiogenous cells, and conidia further corroborates their taxonomic novelty by revealing consistent phenotypic differences from closely related species. Accordingly, four new species of *Microdochium* are proposed: *M. acronychicola*, *M. brevisporum*, *M. chinense*, and *M. wuzhishanense*.

## Introduction

The genus *Microdochium* Syd. & P. Syd. was first described by Sydow (1924) and was placed in *Microdochiaceae* Hern.-Restr., Crous & J.Z. Groenew., *Xylariales* Nannf., *Sordariomycetes* O.E. Erikss. & Winka based on multilocus phylogenetic analyses of ITS, LSU, *RPB2*, and *TUB2* sequences (Hernández-Restrepo et al. 2016; Marin-Felix et al. 2019). Its type species, *Microdochium phragmitis* Syd. & P. Syd., was originally isolated from leaves of *Phragmites australis* (Cav.) Steud. in Germany. To date, 80 nomenclatural records of *Microdochium* have been listed in Index Fungorum (http://www.indexfungorum.org/, accessed on 11 May 2026).

Members of *Microdochium* exhibit distinct morphological characteristics in both sexual and asexual morphs. The sexual morph is *Monographella*-like, characterized by stromatic or astromatic perithecial ascomata, eight-spored oblong to clavate asci with an apical ring, and hyaline to pale brown, fusoid, ellipsoid, or oblong ascospores. The asexual morph is coelomycetous, producing polyblastic, sympodial, or annellidic conidiogenous cells that generate hyaline, falcate, fusiform, or filiform to subpyriform conidia (Hernández-Restrepo et al. 2016). Morphologically, *Microdochium* resembles *Fusarium* but differs in having non-phialidic conidiogenous cells and conidia with truncate rather than foot-shaped basal cells (Gams and Müller 1980; Samuels and Hallett 1983; Logrieco et al. 1991). Historically, its sexual morphs were classified under *Monographella* Petr. (Parkinson et al. 1981; Von Arx 1984; Jaklitsch and Voglmayr 2012), leading to a long-standing polyphyletic circumscription of *Microdochium*/*Monographella*. Hernández-Restrepo et al. (2016) resolved this taxonomic confusion via multigene phylogeny based on the ITS, LSU, *RPB2*, and *TUB2* loci, confirming that *Microdochium* and allied genera form a well-supported monophyletic lineage within *Xylariales*. Following the “one fungus, one name” principle, *Microdochium* was retained as the preferred generic name due to its wider usage in the literature (Hawksworth et al. 2011), and the new family *Microdochiaceae* was established to accommodate *Idriella* P.E. Nelson & S. Wilh., *Microdochium*, and *Selenodriella* R.F. Castañeda & W.B. Kendr. in *Xylariales* (Hernández-Restrepo et al. 2016).

*Microdochium* species exhibit diverse trophic lifestyles, including as phytopathogens, endophytes, and saprobes. Many species act as important plant pathogens, predominantly infecting grasses and cereals (Marin-Felix et al. 2019). For example, *M. majus* (Wollenw.) Glynn & S.G. Edwards and *M. nivale* (Fr.) Samuels & I.C. Hallett are known to inflict considerable harm on cereals and grasses in temperate areas, typically referred to as “*Microdochium* patch” and “pink snow mold” (Von Arx 1987; Glynn et al. 2005; Jewell and Hsiang 2013; Ponomareva et al. 2021). *Microdochium paspali* Wu Zhang bis, Nan & Mei J. Hu was reported to infect leaves of *Paspalum vaginatum* Sw., causing leaf blight (Zhang et al. 2015). *Microdochium poae* J.M. Liang & L. Cai was identified to induce leaf blight in turfgrasses (Liang et al. 2019). *Microdochium seminicola* Hern.-Restr., Seifert, R.M. Clear & B. Dorn was responsible for a new leaf sheath disease on *Zizania latifolia* (Griseb.) Hance ex F.Muell. in China (Yang et al. 2023). Furthermore, some species of *Microdochium* can also exist as endophytes in plant tissues (Mandyam et al. 2010; Shen et al. 2014). Some others behave as saprobes in decaying wood and soil, secreting extracellular enzymes to degrade lignin and cellulose, thereby participating in ecosystem material cycling (Saldajeno et al. 2008; Gao et al. 2022).

Advances in sequencing technology have greatly promoted molecular systematics and species delimitation of fungi. Barr (1990) applied the LSU region to the familial classification of *Monographella*, the teleomorph genus of *Microdochium*, whose taxonomic status was subsequently revised by Lumbsch and Huhndorf (2010). Hong et al. (2008) were the first to use the ITS region for phylogenetic investigations of *Microdochium* species, which preliminarily clarified the phylogenetic relationships among three species of the genus and its type species, thus pioneering molecular systematic research on *Microdochium*. Jewell and Hsiang (2013) made pivotal contributions by designing specific primers for the *RPB2* gene and applying this marker to the molecular systematics of *Microdochium* for the first time, which addressed the limitations of ITS and LSU in distinguishing closely related congeneric species; they also designed primers for the *TUB2* gene and similarly applied this gene to molecular systematic studies of the genus. Building on these foundational single-gene studies, Hernández-Restrepo et al. (2016) integrated the four-gene dataset of ITS, LSU, *RPB2*, and *TUB2* to conduct a comprehensive systematic revision of *Microdochium*, clarifying its generic delimitation, proposing new taxonomic combinations, and establishing the family *Microdochiaceae* to accommodate the genus and its allied taxa. Since then, the four-gene combined analysis has become the standard method for species delimitation and description in *Microdochium*, and subsequent studies from 2023 to 2025 have further expanded the known species diversity and refined the phylogenetic framework of the genus (Zhang et al. 2024; Shang et al. 2025).

Extensive field sampling was undertaken in southern China to investigate the region’s rich fungal diversity and to identify previously undocumented fungal resources. *Microdochium* strains were isolated from diseased leaves of *Bambusoideae* sp., *Acronychia pedunculata* Miq., and *Symplocos sumuntia* Buch.-Ham. ex D. Don, all collected in Hainan Province, China. Through the integration of morphological observations and multilocus phylogenetic analyses, four new species of *Microdochium* are described and illustrated. These findings contribute to the understanding of species diversity and enhance knowledge of the taxonomic and ecological characteristics of this genus.

## Materials and methods

### Sample collection and isolation

The samples in this study were collected in Hainan Province during a series of visits in 2023 and 2024, most of which were diseased or rotted leaves. Collection details were noted (Rathnayaka et al. 2025), and the samples were taken to the laboratory at Shandong Normal University. Although a variety of fungi were present on the leaf samples, pure culture colonies were obtained by single-spore isolation and tissue isolation methods (Wang et al. 2023; Zhang et al. 2025). For single-spore isolation, conidia were evenly spread on water agar, incubated at 25 °C, and individual germinating conidia were picked with a sterile fine needle under a stereomicroscope and transferred onto fresh potato dextrose agar (PDA) medium. Leaf segments of 5 × 5 mm were excised from infected regions of diseased or rotted leaf samples and placed into separate sterile containers. Tissues were surface-disinfected by immersion in 75% ethanol for 1 min, rinsed once with sterile water, then treated with 5% sodium hypochlorite for 30 s, followed by three rinses with sterile water. After drying on sterile filter paper, four leaf pieces were placed symmetrically onto PDA medium (200 g potato, 20 g dextrose, 20 g agar, 1000 mL distilled water, pH 7.0) with lesions facing downward. Plates were sealed, labeled, and incubated at 25 °C. Fungal growth was monitored daily. Hyphal agar blocks from colony margins were transferred to fresh PDA for purification and incubated for 1–2 weeks.

### Morphological and cultural characterization

The characteristics of colonies were photographed on days 7 and 14 with a digital camera (Canon Powershot G7X). Sporulating cultures on PDA media were used to observe the morphological characteristics. Conidial masses on PDA were picked up with a sterile needle and placed on a slide, dripped with a solution of lactic acid-phenol (the ratio of lactic acid, phenol, glycerin, and sterile water was 1:1:2:1), and covered with a coverslip to make a temporary mount. Then, the temporary mount was placed under an Olympus BX53 light microscope furnished with an Olympus DP80 high-definition color digital camera to observe microscopic morphological features, including conidiophores, conidiogenous cells, and conidia. Digimizer software (https://www.digimizer.com/) was used to measure these microstructures at least 15 times for each characteristic. All fungal strains were stored in 10% sterilized glycerin at 4 °C for further studies. The relevant specimens and dry cultures, with conidial masses, from this study were preserved in **HMAS** (Herbarium Mycologicum Academiae Sinicae). The living cultures have been deposited in **SAUCC** (Shandong Agricultural University Culture Collection). Taxonomic information on the new species has been uploaded to MycoBank (http://www.mycobank.org/).

### DNA extraction, PCR amplification, and sequencing

Genomic DNA was extracted from growing colonies on PDA using the CTAB method (Guo et al. 2000). DNA was amplified by polymerase chain reaction (PCR) using the following four loci: ITS, LSU, *RPB2*, and *TUB2*. The primers used in the PCR reaction and the reaction program are shown in Table 1. The amplification reaction was conducted in a 25 μL reaction volume, including 12.5 μL 2× Hieff Canace® Plus PCR Master Mix (Shanghai, China) (with dye) (Yeasen Biotechnology, Shanghai, China, Cat. No. 10154ES03), 1 μL forward primer, 1 μL reverse primer, and 2 μL genomic DNA, with distilled deionized water added to a 25 μL volume. The PCR products were detected by 2% agarose gel electrophoresis, and the amplification was visualized under UV light. Then, a Gel Extraction Kit (Cat. No. AE0101-C; Shandong Sparkjade Biotechnology Co., Ltd.) was used for gel extraction. The purified PCR products were sequenced by Sangon Biotech Co., Ltd. (Shanghai, China). The sequencing data were analyzed and spliced using MEGA v. 7.0 (Kumar et al. 2016). The latest literature on the genus *Microdochium* was reviewed, and GenBank accession numbers for known species were retrieved from the NCBI database, which were then combined with sequences generated in the present study for phylogenetic analyses.

**Table 1.** Gene loci and corresponding PCR primers and programs used in this study.

Locus	PCR primers	Sequence (5’– 3’)	PCR cycles	References
ITS	ITS5	GGA AGT AAA AGT CGT AAC AAG G	(94 °C: 30 s, 55 °C: 30 s, 72 °C: 45 s) × 29 cycles	White et al. (1990)
	ITS4	TCC TCC GCT TAT TGA TAT GC		
LSU	LR0R	GTA CCC GCT GAA CTT AAG C	(94 °C: 30 s, 48 °C: 50 s, 72 °C: 1 min 30 s) × 35 cycles	Vilgalys and Hester (1990)
	LR5	TCC TGA GGG AAA CTT CG		
*RPB2*	RPB2-5F2	GGG GWG AYC AGA AGA AGG C	(94 °C: 45 s, 60 °C: 45 s, 72 °C: 2 min) × 5 cycles, (94 °C: 45 s, 54 °C: 45 s, 72 °C: 2 min) × 30 cycles	Liu et al. (1999); Sung et al. (2007)
	RPB2-7CR	CCC ATR GCT TGY TTR CCC AT		
*TUB2*	Btub526-F	CGA GCG YAT GAG YGT YTA CTT	(95 °C: 30 s, 56 °C: 30 s, 72 °C: 1 min) × 35 cycles	Jewell and Hsiang (2013)
	Btub1332-R	TCA TGT TCT TGG GGT CGA A		

### Phylogenetic analyses

In this study, newly generated sequences were submitted to the NCBI GenBank database. The reference sequences used in this study were obtained from the National Center for Biotechnology Information (NCBI) according to the most recent publication on *Microdochium*. Sequences newly generated in this study were aligned with relevant sequences obtained from GenBank using the online MAFFT 7 tool with the Auto strategy (Katoh et al. 2019), followed by manual refinement in BioEdit (Hall 2006). Phylogenetic analysis of multilocus data was conducted using Bayesian inference (BI) and maximum likelihood (ML) algorithms. First, MrModeltest v. 2.3 was employed to screen for the optimal evolutionary model—specifically, to determine the best evolutionary model for each partition under the Akaike Information Criterion (AIC), which identified the optimal nucleotide substitution model settings before BI analysis. Second, ML and BI were run on the CIPRES Science Gateway portal (https://www.phylo.org/, accessed on 22 December 2025) or via offline software (Miller et al. 2012). ML was performed using the RAxML-HPC2 tool on XSEDE (version 8.2.12), with 1,000 fast/rapid bootstrap replicates under the GTRGAMMA nucleotide model and default parameters (Stamatakis 2014). BI was carried out using XSEDE (version 3.2.7a), adopting the Markov chain Monte Carlo (MCMC) algorithm, and the phylogenetic topology was output when the mean standard deviation of split frequencies was less than 0.01 (Ronquist and Huelsenbeck 2003; Ronquist et al. 2012). Finally, all generated trees were plotted using FigTree v. 1.4.4 (http://tree.bio.ed.ac.uk/software/figtree, accessed on 28 December 2025) and iTOL: Interactive Tree of Life (https://itol.embl.de/, accessed on 30 December 2025), and their layout was refined and produced in Adobe Illustrator CC 2023, with the names of the isolates in this study marked in red in the phylogenetic trees (Letunic and Bork 2021). The species information used in this research is presented in Table 2.

**Table 2.** GenBank accession numbers of the taxa in the phylogenetic reconstruction.

Species	Culture accession	GenBank accession numbers				Reference
		ITS	LSU	*TUB2*	*RPB2*	
***Microdochium acronychicola***	SAUCC620701*	**PV390632**	**PV933123**	**PX393495**	**PV943943**	**This study**
***M. acronychicola***	**SAUCC620702**	**PV390633**	**PV933124**	**PX393496**	**PV943944**	**This study**
*M. albescens*	CBS 243.83	KP858994	KP858930	KP859057	KP859103	Hernández-Restrepo et al. (2016)
*M. albescens*	CBS 291.79	KP858996	KP858932	KP859059	KP859105	Hernández-Restrepo et al. (2016)
*M. australiana*	SAUCC6340-2-6	PQ807110	PV609100	PV686755	PV975978	Shang et al. (2025)
*M. australiana*	SAUCC8723-2	PQ807111	PV609101	PV686756	PV975979	Shang et al. (2025)
*M. bambusae*	SAUCC 1862–1	OR702567	OR702576	OR715791	OR715785	Zhang et al. (2023)
*M. bambusae*	SAUCC 1866–1	OR702568	OR702577	OR715792	OR715786	Zhang et al. (2023)
*M. bambusinum*	SAUCC7531-3	PQ807108	PV609098	PV686753	PV975976	Shang et al. (2025)
*M. bambusinum*	SAUCC7638-2	PQ807109	PV609099	PV686754	PV975977	Shang et al. (2025)
*M. bambusarum*	SAUCC7611-3	PQ807112	PV609102	PV686757	PV975980	Shang et al. (2025)
*M. bambusarum*	SAUCC6699-4	PQ807113	PV609103	PV686758	PV975981	(Shang et al. 2025)
*M. baishamenense*	SAUCC8129-1	PQ807114	PV609104	PV686759	PV975982	Shang et al. (2025)
*M. baishamenense*	SAUCC7263-1	PQ807115	PV609105	PV686760	PV975983	Shang et al. (2025)
*M. bolleyi*	CBS 540.92	KP859010	KP858946	KP859073	KP859119	Hernández-Restrepo et al. (2016)
***M. brevisporum***	SAUCC785206*	**PV390634**	**PV933125**	**PX393497**	**–**	**This study**
***M. brevisporum***	**SAUCC785207**	**PV390635**	**PV933126**	**PX393498**	**–**	**This study**
***M. chinense***	SAUCC1516001*	**PV390636**	**PV933127**	**PX393499**	**PV943945**	**This study**
***M. chinense***	**SAUCC1516002**	**PV390637**	**PV933128**	**PX393500**	**PV943946**	**This study**
*M. chrysanthemoides*	LC5363*	KU746690	KU746736	KU746781	KY883244	Zhang et al. (2017)
*M. chrysanthemoides*	LC5466	KU746689	KU746735	KU746782	KY883245	Zhang et al. (2017)
*M. chrysopogonis*	GDMCC 3.683	MT988022	MT988024	MW002441	MW002444	(Lu et al. 2023)
*M. chuxiongense*	YFCC 8794	OK586161	OK586160	OK556901	OK584019	Tang et al. (2022)
*M. citrinidiscum*	CBS 109067*	KP859003	KP858939	KP859066	KP859112	Hernández-Restrepo et al. (2016)
*M. colombiense*	CBS 624.94*	KP858999	KP858935	KP859062	KP859108	Hernández-Restrepo et al. (2016)
*M. dawsoniorum*	BRIP 65649*	MK966337	ON394569	–	–	Crous et al. (2020)
*M. dawsoniorum*	BRIP 67439a	MN492650	OM333563	–	ON624208	Crous et al. (2020)
*M. fisheri*	CBS 242.90	KP859015	KP858951	KP859079	KP859124	Hernández-Restrepo et al. (2016)
*M. guizhouensis*	GUCC 25–0012	PV487384	PV487386	PV505433	PV505431	Hongsanan et al. (2025)
*M. guizhouensis*	GUCC 25 0013	PV487385	PV487387	PV505434	PV505432	Hongsanan et al. (2025)
*M. graminearum*	CGMCC 3.23524	OP103965	OP104015	OP242835	OP236026	Gao et al. (2022)
*M. graminearum*	CGMCC 3.23525*	OP103966	OP104016	OP236029	OP236027	Gao et al. (2022)
*M. graminis*	ZJE01778*	PP111928	PP111935	–	–	Yan and Zhang (2024)
*M. graminis*	B13	HQ696038	–	–	–	Shen et al. (2014)
*M. graminis*	PE110	JX875927	–	–	–	Shen et al. (2012)
*M. gongcheniae*	YNE01155	PP111925	PP111932	–	–	Yan and Zhang (2024)
*M. gongcheniae*	YNE01164*	PP111926	PP111933	–	–	Yan and Zhang (2024)
*M. hainanense*	SAUCC210781 *	OM956295	OM959323	OM981146	OM981153	Liu et al. (2022)
*M. hainanense*	SAUCC210782	OM956296	OM959324	OM981147	OM981154	Liu et al. (2022)
*M. hongkuii*	YNE00384	PP111922	PP111929	PP112582	PP112589	Yan and Zhang (2024)
*M. hongkuii*	YNE00483*	PP111923	PP111930	PP112583	PP112590	Yan and Zhang (2024)
*M. hongkuii*	N115	MK304137	–	–	–	From NCBI
*M. indocalami*	SAUCC1016 *	MT199884	MT199878	MT435653	MT510550	Huang et al. (2020)
*M. insulare*	BRIP 75114a	OQ917075	OQ892168	–	OQ889560	Tan and Shivas (2023a)
*M. lycopodinum*	CBS 109397	KP859004	KP858940	KP859067	KP859113	Hernández-Restrepo et al. (2016)
*M. lycopodinum*	CBS 109398	KP859005	KP858941	KP859068	KP859114	Hernández-Restrepo et al. (2016)
*M. lycopodinum*	CBS 125585 *	KP859016	KP858952	KP859080	KP859125	Hernández-Restrepo et al. (2016)
*M. maculosum*	COAD 3358 *	OK966954	OK966953	–	OL310501	Crous et al. (2021a, 2021b)
*M. majus*	CBS 741.79	KP859001	KP858937	KP859064	KP859110	Hernández-Restrepo et al. (2016)
*M. miscanthi*	SAUCC211092 *	OM956214	OM957532	OM981141	OM981148	Liu et al. (2022)
*M. miscanthi*	SAUCC211093	OM956215	OM957533	OM981142	OM981149	Liu et al. (2022)
*M. musae*	CBS 143499	MH107894	MH107941	MH108040	–	Crous et al. (2018)
*M. musae*	CBS 143500 *	MH107895	MH107942	MH108041	MH108003	Crous et al. (2018)
*M. nannuoshanense*	SAUCC 2450–1*	OR702569	OR702578	OR715793	OR715787	Zhang et al. (2023)
*M. nannuoshanense*	SAUCC 2450–3	OR702570	OR702579	OR715794	OR715788	Zhang et al. (2023)
*M. neoqueenslandicum*	CBS 108926*	KP859002	KP858938	KP859065	KP859111	Hernández-Restrepo et al. (2016)
*M. neoqueenslandicum*	CBS 445.95	KP858997	KP858933	KP859060	KP859106	Hernández-Restrepo et al. (2016)
*M. nivale*	CBS 116205*	KP859008	KP858944	KP859071	KP859117	Hernández-Restrepo et al. (2016)
*M. nivale*	CBS 288.50	MH856626	MH868135	–	–	Vu et al. (2019)
*M. novae-zelandiae*	CBS 143847 *	LT990655	LT990627	LT990608	LT990641	Marin-Felix et al. (2019)
*M. novae-zelandiae*	CPC 29693	LT990656	LT990628	LT990609	LT990642	Marin-Felix et al. (2019)
*M. oryzicola*	MFLUCC 24-0509	PV241406	PV241407	–	PV275683	Absalan et al. (2025)
*M. paspali*	CBS 138620*	KJ569513	–	KJ569518	–	Zhang et al. (2015)
*M. paspali*	QH-BA-48	KJ569510	–	KJ569515	–	Zhang et al. (2015)
*M. phyllosaprophyticum*	SAUCC 3583–1*	OR702571	OR702580	OR715795	OR715789	Zhang et al. (2023)
*M. phyllosaprophyticum*	SAUCC 3583–6	OR702572	OR702581	OR715796	OR715790	Zhang et al. (2023)
*M. phragmitis*	CBS 285.71 *	KP859013	KP858949	KP859077	KP859122	Hernández-Restrepo et al. (2016)
*M. phragmitis*	CBS 423.78	KP859012	KP858948	KP859076	KP859121	Hernández-Restrepo et al. (2016)
*M. poae*	LC12114 *	MH740898	–	MH740914	MH740906	Liang et al. (2019)
*M. ratticaudae*	BRIP 68298 *	MW481661	MW481666	–	MW626890	Crous et al. (2021a, 2021b)
*M. rhopalostylidis*	CBS 145125 *	MK442592	MK442532	MK442735	MK442667	Crous et al. (2019)
*M. salmonicolor*	KCTC 56427	NR173378	MK836108	–	–	Das et al. (2020)
*M. seminicola*	KAS1516	KP859025	KP858961	KP859088	KP859134	Hernández-Restrepo et al. (2016)
*M. seminicola*	KAS3576*	KP859038	KP858974	KP859101	KP859147	Hernández-Restrepo et al. (2016)
*M. shilinense*	CGMCC 3.23531 *	OP103972	OP104022	OP242834	–	Gao et al. (2022)
*M. sichuanense*	KUNCC23-13008 *	OQ616510	OQ616434	–	OQ623473	Dissanayake et al. (2023)
*M. sinense*	SAUCC211097 *	OM956289	OM959225	OM981144	OM981151	Liu et al. (2022)
*M. sinense*	SAUCC211098	OM956290	OM959226	OM981145	OM981152	Liu et al. (2022)
*M. sorghi*	CBS 691.96	KP859000	KP858936	KP859063	KP859109	Hernández-Restrepo et al. (2016)
*Microdochium* sp.	YNE01043	PP111924	PP111931	PP112584	PP112591	Yan and Zhang (2024)
*Microdochium* sp.	YNE01771	PP111927	PP111934	–	PP112592	Yan and Zhang (2024)
*Microdochium* sp.	ZJ40	KJ572190	–	–	–	From NCBI
*M. streetiae*	BRIP 74742a*	OR947072	OR947079	–	–	Tan and Shivas (2023b)
*M. streetiae*	BRIP 74752a	OR947071	OR947078	–	–	Tan and Shivas (2023b)
*M. tainanense*	CBS 269.76 *	KP859009	KP858945	KP859072	KP859118	Hernández-Restrepo et al. (2016)
*M. tainanense*	CBS 270.76	KP858995	KP858931	KP859058	KP859104	Hernández-Restrepo et al. (2016)
*M. trichocladiopsis*	CBS 623.77 *	KP858998	KP858934	KP859061	KP859107	Vu et al. (2019)
*M. triticicola*	RR 241	AJ748691	–	–	–	Kwasna and Bateman (2007)
***M. wuzhishanense***	SAUCC14246-4 *	**PX745087**	**PX752258**	**PX753105**	**PX755900**	**This study**
***M. wuzhishanense***	**SAUCC14246-4b**	**PX745088**	**PX752259**	**PX753106**	**PX755901**	**This study**
*M. yunnanense*	SAUCC1011*	MT199881	MT199875	MT435650	MT510547	Huang et al. (2020)
*M. yunnanense*	SAUCC1015	MT199883	MT199877	MT435652	MT510549	Huang et al. (2020)
*Muscodor fengyangensis*	CGMCC 2862*	HM034856	HM034859	HM034843	HM034849	Zhang et al. (2010)
*M. thailandicus*	MFLUCC 17–2669*	MK762707	MK762714	MK776960	MK791283	Samarakoon et al. (2020)
*Peglionia verticiclada*	CBS 127654*	ON400763	ON400815	–	ON399352	Hernández-Restrepo et al. (2022)
*Selenodriella brasiliana*	CBS 140227*	ON400769	ON400821	–	ON399356	Hernández-Restrepo et al. (2022)
*S. cubensis*	CBS 683.96*	KP859053	KP858990	–	–	Hernández-Restrepo et al. (2016)
*S. fertilis*	CBS 772.83	KP859055	KP858992	–	–	Hernández-Restrepo et al. (2016)
*S. fertilis*	CPC 16273	ON400771	ON400823	–	ON399358	Crous et al. (2019)

Notes: New species established in this study are shown in bold. Those marked “*” in the table are represented as ex-type or ex-epitype strains. “-”: Not available.

## Results

### Phylogenetic analyses

**Figure 1.**
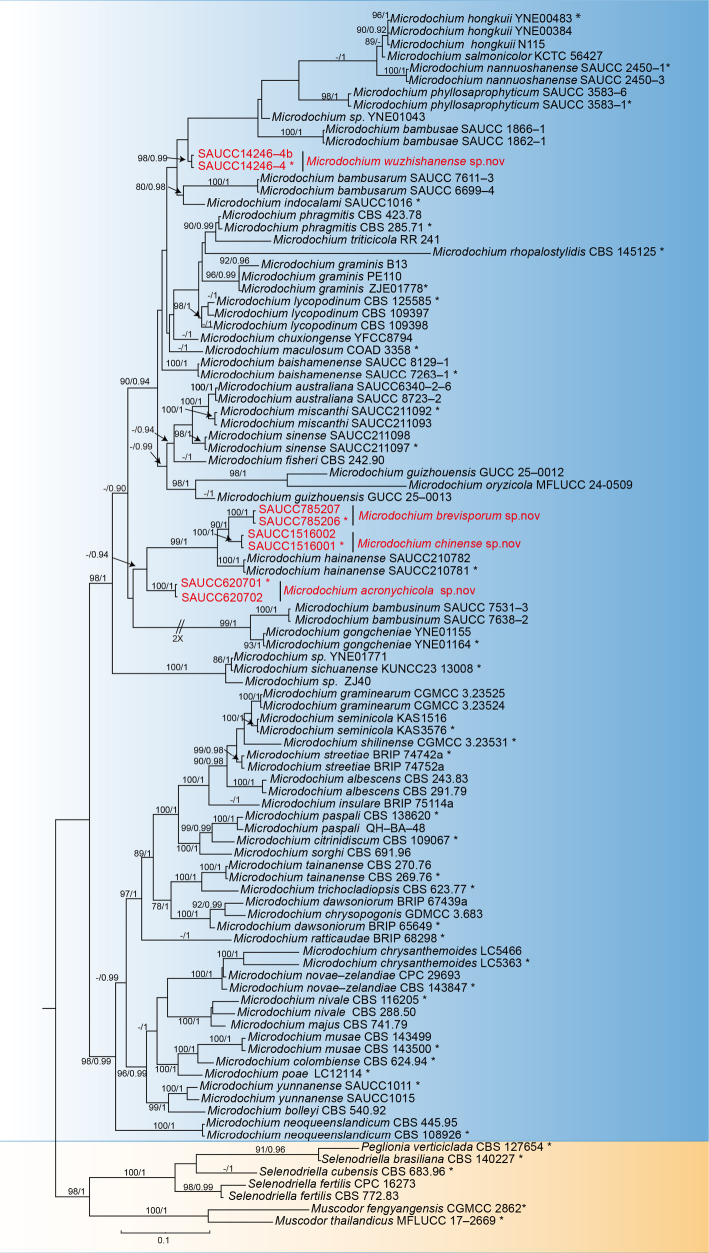
Maximum likelihood inference tree based on a combined dataset of analyzed ITS, LSU, *RPB2*, and *TUB2* sequences. The maximum likelihood bootstrap value (left, MLBS ≥ 70%) and the Bayesian inference posterior probability (right, BYPP ≥ 0.90) are shown as MLBS/BYPP above the nodes. Those marked “*” in the tree are represented as ex-type or ex-epitype strains. “*Microdochium*” text in blue box and “outgroup” text in yellow box. Strains isolated in this study are indicated in red. The scale bar at the bottom indicates 0.1 substitutions per site. To enhance the visual appeal of the evolutionary tree layout, certain branches are shortened by two diagonal lines (“//”) with the number of times.

By analyzing ITS, LSU, *RPB2*, and *TUB2* sequence datasets, interspecific relationships within *Microdochium* were identified. The phylogenetic analyses contained 99 sequences, using *Muscodor fengyangensis* (CGMCC 2862), *M. thailandicus* (MFLUCC 17-2669), and sequences of *Peglionia verticiclada* (CBS 127654), *Selenodriella brasiliana* (CBS 140227), *S. cubensis* (CBS 683.96), and *S. fertilis* (CBS 772.83, CPC 16273) as outgroups. A total of 3,611 characters, including gaps, were included in the phylogenetic analysis, viz. 1–727 for ITS, 728–1,599 for LSU, 1,600–2,595 for *RPB2*, and 2,596–3,611 for *TUB2*. Of all characters, 2,036 characters were constant, while 316 variable characters were parsimony-uninformative, and the number of parsimony-informative characters was 1,259. In Bayesian analysis, the GTR+I+G model was proposed for ITS and *RPB2*, the SYM+I+G model for LSU, and the GTR+G model for *TUB2*. Phylogenetic trees generated via the maximum likelihood method and Bayesian inference algorithm shared similar topological structures; thus, they can be considered to represent the evolutionary history of *Microdochium*. The best-scoring maximum likelihood evolutionary tree, where maximum likelihood bootstrap analyses and Bayesian posterior probabilities are labeled at node positions, is shown in Fig. 1. In total, the 99 strains analyzed were assigned to 62 distinct species. Within the phylogenetic tree, the eight isolates obtained in the present study were clustered into four independent, highly supported monophyletic clades, each of which was distinctly separated from all previously described *Microdochium* taxa. Specifically, each clade contained two isolates with obvious interspecific genetic divergence, indicating that these four lineages represented four species of *Microdochium*. In terms of phylogenetic affinity, *Microdochium wuzhishanense* (SAUCC14246-4 and SAUCC14246-4b) was closely related to *M. bambusae* (SAUCC1866-1 and SAUCC1862-1) and *M. bambusarum* (SAUCC7611-3 and SAUCC6699-4). *Microdochium chinense* (SAUCC1516001 and SAUCC1516002) formed a sister lineage to *M. brevisporum* (SAUCC785206 and SAUCC785207) with MLBS/BYPP = 90/1 statistical support. Meanwhile, the clade containing these two species formed a major sister clade with the clade of *M. hainanense* (SAUCC210781 and SAUCC210782) with MLBS/BYPP = 99/1 statistical support. The clade comprising the above three species formed a larger sister clade with the clade of *M. acronychicola* (SAUCC620701 and SAUCC620702) with MLBS/BYPP = –/0.94 statistical support. The relatively low MLBS (< 70) may be attributed to recent rapid speciation among closely related taxa, leading to insufficient nucleotide variation and limited parsimony-informative sites in the concatenated dataset. Phylogenetic signal heterogeneity among the four genes, especially low sequence divergence in the conserved LSU region, may also reduce the resolution of the ML tree at this node. However, the corresponding nodes in the BI tree received high posterior probability support, indicating that the relationships are reliably supported by the multigene data.

### Taxonomy

#### Microdochium acronychicola

Taxon classificationFungiAmphisphaerialesAmphisphaeriaceae

X.G. Yan, Y.J. Wang, C.C. Ai & X.G. Zhang sp. nov.

73F2BA6D-913E-5A90-96AF-8D0DC11E7E94

861251

Fig. 2

##### Type.

China • Hainan Province: Ledong Li Autonomous County, on diseased leaves of *Acronychia pedunculata* Miq., 13 October 2023, X.G. Yan (HMAS 354067, holotype), ex-type living culture SAUCC620701 = SAUCC620702.

**Figure 2.**
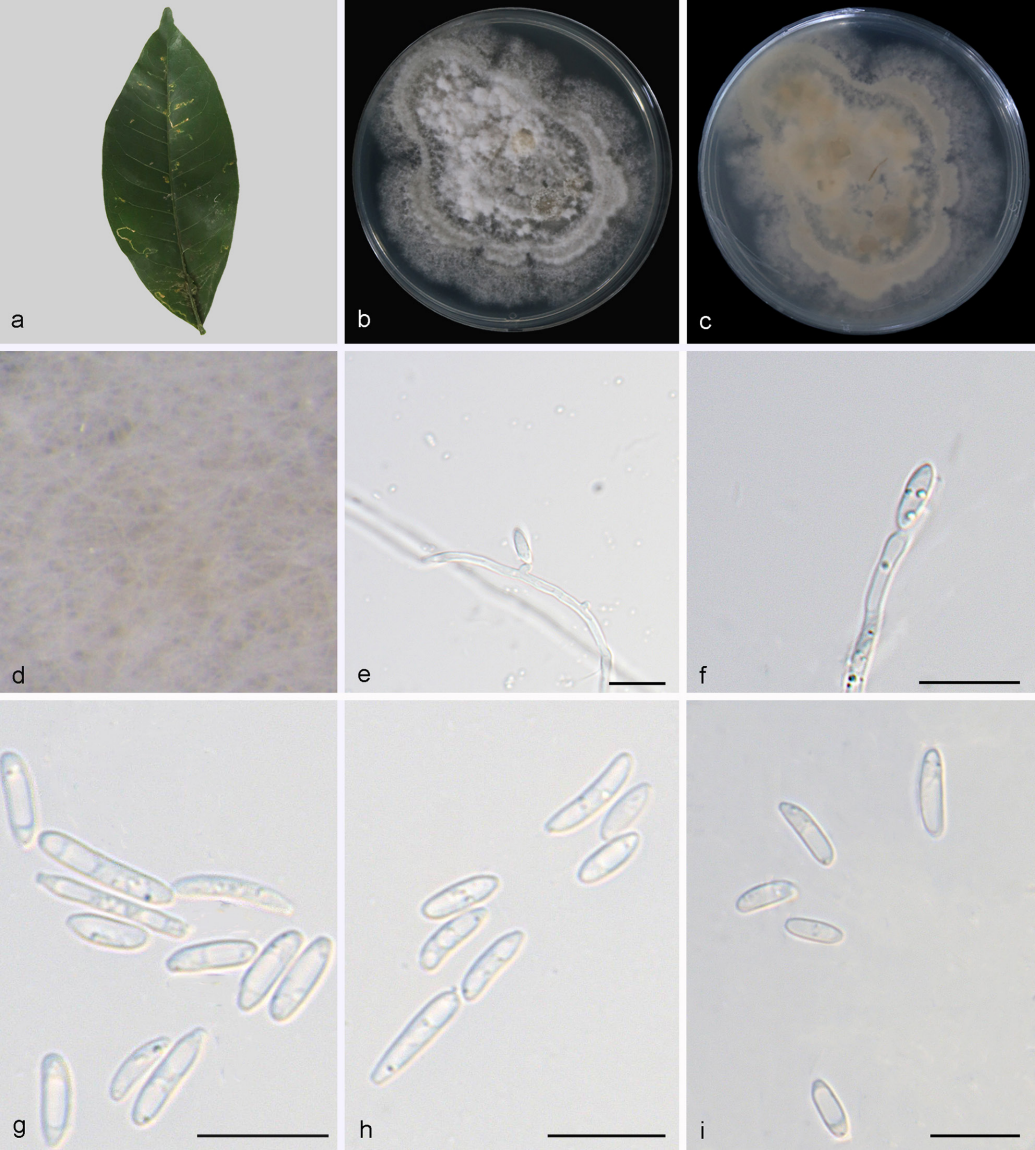
*Microdochium acronychicola* (SAUCC620701, ex-type). **a**. A diseased leaf of *Acronychia pedunculata*; **b, c**. Colony on PDA from above and below after 14 days; **d**. Colony overview; **e, f**. Conidiogenous cells with conidia; **g–i**. Conidia. Scale bars: 10 μm (**e–i**).

##### Etymology.

Refers to the genus *Acronychia*, from which the fungus was isolated.

##### Description.

Associated with diseased leaves of *Acronychia pedunculata*. **Asexual morph on PDA: *Mycelia*** superficial, membranous and immersed, hyaline, branched, septate, and smooth, 1.1–2.8 μm wide (x̄ = 1.77 μm, *n* = 30). ***Conidiomata*** not observed. ***Conidiophores*** indistinct and always reduced to conidiogenous cells. ***Conidiogenous cells*** polyblastic, hyaline, straight or slightly curved, smooth-walled, arising from apices of branched hyphae, 1.4–3.7 × 1.2–2.0 μm (x̄ = 2.4 × 1.6 μm, *n* = 15). ***Conidia*** solitary, smooth-walled, elliptical or fusiform, septate, 2–3-septate, and straight or slightly curved, 5.4–11.6 × 1.5–2.7 μm (x̄ = 7.1 × 2.1 μm, *n* = 65). ***Chlamydospores*** not observed. **Sexual morph**: Undetermined.

##### Culture characteristics.

Colonies were incubated on PDA medium at 25 °C under dark conditions. After 14 days of cultivation, the colonies attained a diameter of 65–87 mm, with a daily growth rate ranging from 4.6 to 6.2 mm. Colonies exhibited an irregular outward growth pattern, covered with abundant white aerial mycelium. With prolonged incubation, the central region of the colony gradually changed from white to yellowish-brown, and the pigmentation subsequently extended toward the peripheral areas. The reverse surface was white at the initial stage and ultimately turned orange. Sparse sporulation was observed on PDA after 14 days of incubation.

##### Notes.

Based on phylogenetic analysis combined with four sequences (ITS, LSU, *RPB2*, and *TUB2*), *Microdochium acronychicola* (SAUCC620701 and SAUCC620702) constitutes an independent clade in the phylogenetic trees, closely related to *M. hainanense* (SAUCC210781 and SAUCC210782) and *M. bambusinum* (SAUCC7638-2 and SAUCC7531-3). *Microdochium acronychicola* (SAUCC620701, ex-type) is distinguished from *M. hainanense* (SAUCC210781, ex-type) by 18/558, 1/805, 62/976, and 35/753 characters in ITS, LSU, *RPB2*, and *TUB2* sequences, respectively, with all gap sites excluded from the sequence comparisons. *Microdochium acronychicola* (SAUCC620701, ex-type) is distinguished from *M. bambusinum* (SAUCC7638-2, ex-type) by 18/548, 11/806, 89/897, and 93/767 characters in ITS, LSU, *RPB2*, and *TUB2* sequences, respectively, with all gap sites excluded from the sequence comparisons. Morphologically, the conidia of *M. acronychicola* (HMAS 354067, holotype) are shorter than those of *M. hainanense* (HMAS 352156, holotype) S.B. Liu, X.Y. Liu, Z. Meng & X.G. Zhang and longer than those of *M. bambusinum* (HSAUP 7531-3, holotype) (5.4–11.6 × 1.5–2.7 μm vs. 11.5–19.34 × 2.8–5.4 µm vs. 4.8–7.2 × 2.1–3.4 µm). It was also found that the collection of *M. acronychicola* (SAUCC620701 and SAUCC620702) is closely related to two other species in this collection, namely *M. chinense* (SAUCC1516001 and SAUCC1516002) and *M. brevisporum* (SAUCC785206 and SAUCC785207). *Microdochium acronychicola* (SAUCC620701, ex-type) is distinguished from *M. chinense* (SAUCC1516001, ex-type) by 4/550, 3/810, 16/974, and 25/750 characters in ITS, LSU, *RPB2*, and *TUB2* sequences, respectively, with all gap sites excluded from the sequence comparisons. *Microdochium acronychicola* (SAUCC620701, ex-type) is distinguished from *M. brevisporum* (SAUCC785206, ex-type) by 19/554, 16/788, and 50/733 characters in ITS, LSU, and *TUB2* sequences, respectively, with all gap sites excluded from the sequence comparisons. Morphologically, the conidiogenous cells of *M. acronychicola* (HMAS 354067, holotype) are shorter than those of *M. chinense* (HMAS 354069, holotype) and *M. brevisporum* (HMAS 354068, holotype) (1.4–3.7 × 1.2–2.0 μm vs. 4.2–22.9 × 1.7–2.3 μm vs. 1.4–9.6 × 0.9–3.0 μm). The conidia of *M. acronychicola* (HMAS 354067, holotype) are shorter than those of *M. chinense* (HMAS 354069, holotype) and longer than those of *M. brevisporum* (HMAS 354068, holotype) (5.4–11.6 × 1.5–2.7 μm vs. 5.1–13.8 × 1.5–2.9 μm vs. 4.6–9.1 × 1.7–4.3 μm). Therefore, based on morphology and phylogeny, this fungus is established as *Microdochium acronychicola* sp. nov.

#### Microdochium brevisporum

Taxon classificationFungiAmphisphaerialesAmphisphaeriaceae

X.G. Yan, Y.J. Wang, C.C. Ai & X.G. Zhang sp. nov.

ACF6CADA-1AA8-5AD7-B957-47BABA5CE2B4

863344

Fig. 3

##### Type.

China • Hainan Province: Wuzhishan National Natural Reserve, on diseased leaves of *Bambusoideae* sp., 28 March 2024, X.G. Yan (HMAS 354068, holotype), ex-type living culture SAUCC785206 = SAUCC785207.

**Figure 3.**
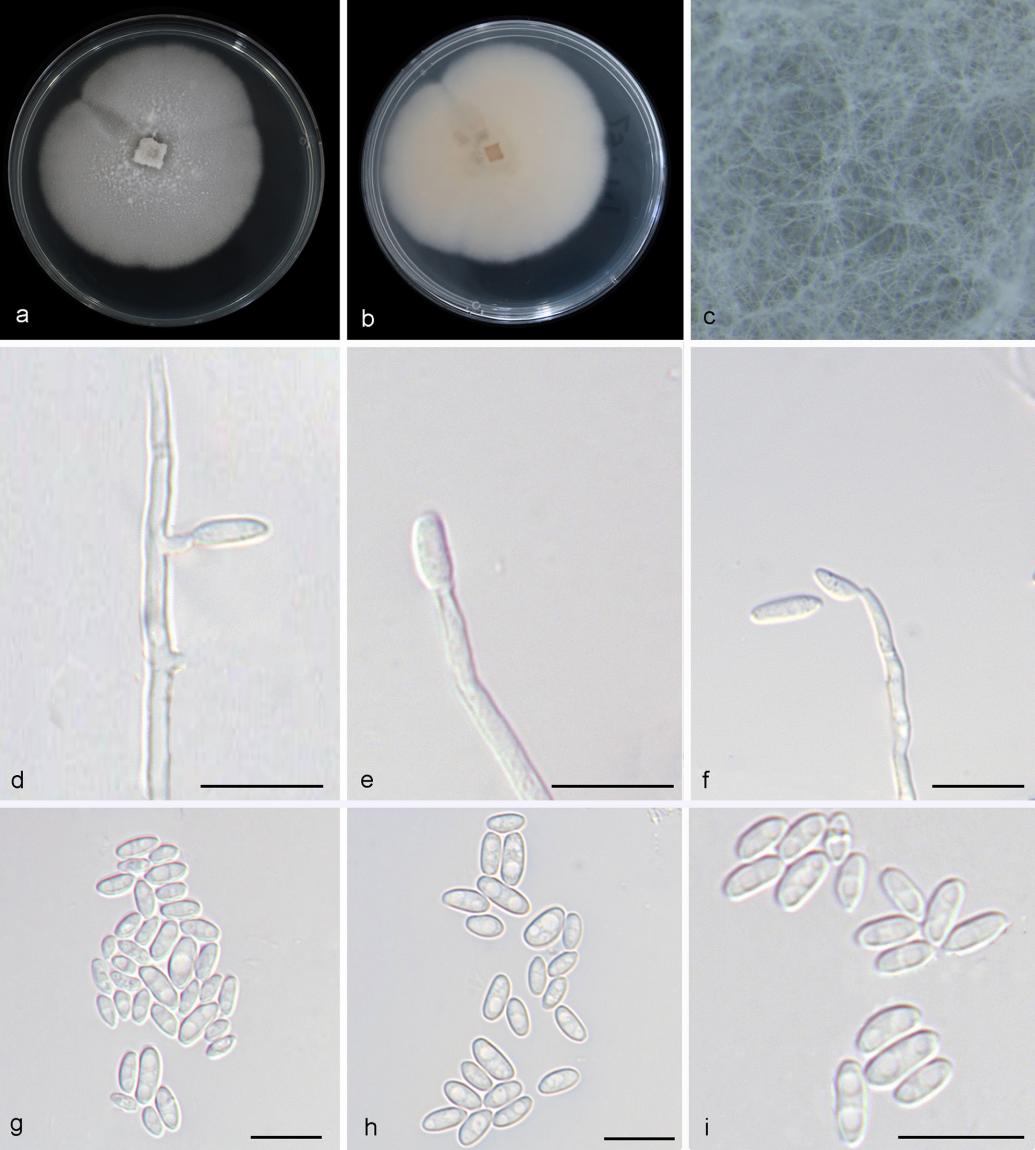
*Microdochium brevisporum* (SAUCC 785206, ex-type). **a, b**. Colony on PDA from above and below after 14 days; **c**. Colony overview; **d–f**. Conidiogenous cells with conidia; **g–i**. Conidia. Scale bars: 10 μm (**d–i**).

##### Etymology.

brevis – (Latin) meaning short + sporum (Latin) meaning spored, refers to the relatively short conidia of this fungus.

##### Description.

Associated with diseased leaves of *Bambusoideae* sp. **Asexual morph on PDA: *Mycelium*** immersed and superficial, hyaline, branched, septate, smooth, 1.1–2.5 μm wide (x̄ = 1.59 μm, *n* = 30). ***Conidiophores*** cylindrical, branched, arising from hyphae, often reduced to conidiogenous cells. ***Conidiogenous cells*** polyblastic, hyaline, straight or occasionally curved, smooth-walled, 1.4–9.6 × 0.9–3.0 μm (x̄ = 4.6 × 1.5 μm, *n* = 20). ***Conidia*** solitary or branched, hyaline, elliptical to subcylindrical, smooth-walled, straight or slightly curved, 0–2-septate, 4.6–9.1 × 1.7–4.3 μm (x̄ = 6.0 × 2.4 μm, *n* = 75). ***Chlamydospores*** not observed. **Sexual morph**: Undetermined.

##### Culture characteristics.

Colonies incubated on PDA at 25 °C under dark conditions attained 63–71 mm in diameter after 14 days, with a daily growth rate ranging from 4.5 to 5.1 mm. Colonies were overall white and exhibited concentric zonate growth radiating from the center. At 14 days of incubation, the aerial surface remained persistently white; the reverse surface gradually turned brown in the central region, and sporogenous structures were progressively produced in the central colony area. Sporulation commenced on PDA after 21 days of incubation, with conidia formed directly on vegetative hyphae.

##### Notes.

Phylogenetic analyses combined with four gene sequences (ITS, LSU, *RPB2*, and *TUB2*) showed that *Microdochium brevisporum* (SAUCC785206 and SAUCC785207) constitutes an independent clade, closely related to *M. hainanense* (SAUCC210782 and SAUCC210781) and *M. chinense* (SAUCC1516001 and SAUCC1516002). *Microdochium brevisporum* (SAUCC785206, ex-type) is distinguished from *M. hainanense* (SAUCC210781, ex-type) by 22/557, 6/788, and 40/733 characters in ITS, LSU, and *TUB2* sequences, respectively, excluding gap sites. *Microdochium brevisporum* (SAUCC785206, ex-type) is distinguished from *M. chinense* (SAUCC1516001, ex-type) by 15/552, 6/788, and 53/733 characters in ITS, LSU, and *TUB2* sequences, respectively, excluding gap sites. Morphologically, the conidia of *M. brevisporum* (HMAS 354068, holotype) are shorter than those of *M. hainanense* (HMAS 352156, holotype) and *M. chinense* (HMAS 354069, holotype) (4.6–9.1 × 1.7–4.3 μm vs. 11.5–19.34 × 2.8–5.4 µm vs. 5.1–13.8 × 1.5–2.9 μm). The conidiogenous cells of *M. brevisporum* (HMAS 354068, holotype) are shorter than those of *M. hainanense* (HMAS 352156, holotype) and *M. chinense* (HMAS 354069, holotype) (1.4–9.6 × 0.9–3.0 μm vs. 16.3–22.4 × 4.1–5.7 µm vs. 4.2–22.9 × 1.7–2.3 μm). In addition, the colonies of *M. brevisporum* (HMAS 354068, holotype) appear white overall and grow from the center in a concentric ring-like pattern, while the colonies of *M. chinense* (HMAS 354069, holotype) are predominantly white, with some areas appearing pale yellowish (Liu et al. 2022). Therefore, based on morphology and phylogeny, this fungus is established as *Microdochium brevisporum* sp. nov.

#### Microdochium chinense

Taxon classificationFungiAmphisphaerialesAmphisphaeriaceae

X.G. Yan, Y.J. Wang, C.C. Ai & X.G. Zhang sp. nov.

56F76904-20D4-5B41-A95E-15967DAF2BDE

861252

Fig. 4

##### Type.

China • Hainan Province: Wuzhishan National Natural Reserve, on diseased leaves of *Bambusoideae* sp., 3 December 2024, X.G.Yan (HMAS 354069, holotype), ex-type living culture SAUCC1516001 = SAUCC1516002.

**Figure 4.**
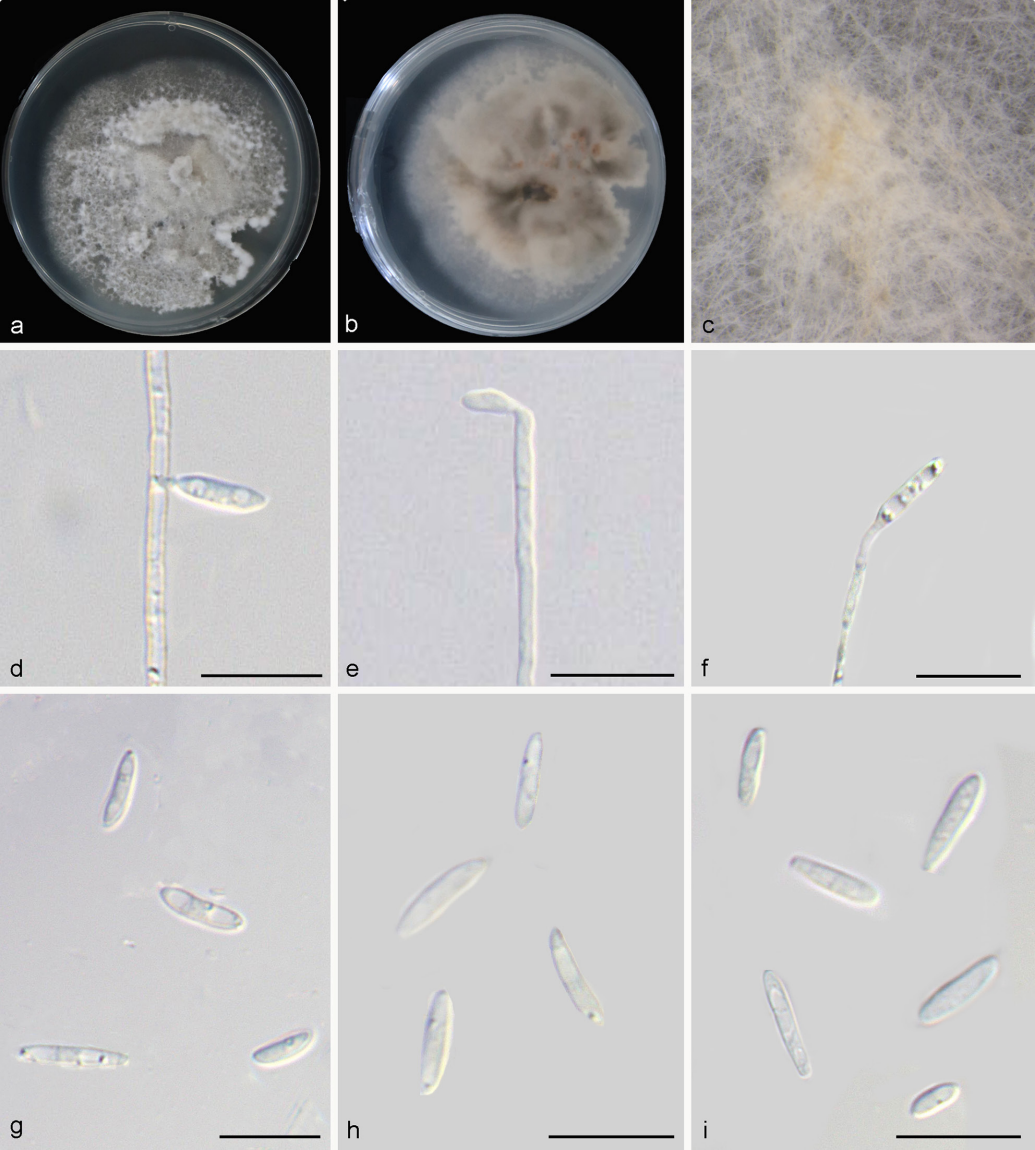
*Microdochium chinense* (SAUCC1516001, ex-type). **a, b**. Above and below sides of colony after 14 days on PDA; **c**. Colony overview with conidiomata; **d–f**. Conidiogenous cells with conidia; **g–i**. Conidia. Scale bars: 10 μm (**d–i**).

##### Etymology.

Refers to the type locality, China.

##### Description.

Associated with diseased leaves of *Bambusoideae* sp. **Asexual morph on PDA: *Mycelia*** superficial and immersed, hyaline, branched, septate, smooth, 1.4–2.8 μm wide (x̄ = 2.03 μm, *n* = 50). ***Conidiophores*** inconspicuous, often reduced to conidiogenous cells. ***Conidiogenous cells*** polyblastic, cylindrical, hyaline and smooth, straight or slightly curved, arise from the tips or from the sides of the hyphae, 4.2–22.9 × 1.7–2.3 μm (x̄ = 13.3 × 2.1 μm, *n* = 15). ***Conidia*** hyaline, smooth-walled, subfalcate to elliptical, 0–3-septate, 5.1–13.8 × 1.5–2.9 μm (x̄ = 8.2 × 2.4 μm, *n* = 55). ***Chlamydospores*** not observed. **Sexual morph**: Undetermined.

##### Culture characteristics.

Colonies grown on PDA at 25 °C in darkness reached 62–72 mm in diameter after 14 days, with a daily growth rate of 4.4–5.1 mm. Colonies grew radially from the center with an approximately circular outline. The aerial surface was predominantly white, with partial areas fading to pale yellowish. The reverse surface was black at the center, gradually fading outwards in a graded color transition from brown to greyish-brown. Colony margins exhibited uneven growth and remained white; the overall reverse pigmentation was brownish, with scattered pale orange regions observable within the colony. Sporulation commenced on PDA after 21 days of incubation, with conidia formed in slimy masses on the aerial mycelium.

##### Notes.

Phylogenetic analysis of four combined gene sequences (ITS, LSU, *RPB2*, and *TUB2*) showed that *Microdochium chinense* (SAUCC1516001 and SAUCC1516002) is closely affiliated with *M. brevisporum* (SAUCC785206 and SAUCC785207) and *M. hainanense* (SAUCC210781 and SAUCC210782). *Microdochium chinense* (SAUCC1516001, ex-type) is distinguished from *M. brevisporum* (SAUCC785206, ex-type) by 15/552, 6/788, and 53/733 characters in ITS, LSU, and *TUB2* sequences, respectively, excluding gap sites. *Microdochium chinense* (SAUCC1516001, ex-type) is distinguished from *M. hainanense* (SAUCC210781, ex-type) by 15/555, 36/857, 66/976, and 40/750 characters in ITS, LSU, *RPB2*, and *TUB2* sequences, respectively, excluding gap sites. Morphologically, the conidia of *M. chinense* (HMAS 354069, holotype) are shorter than those of *M. hainanense* (HMAS 352156, holotype) and longer than those of *M. brevisporum* (HMAS 354068, holotype) (5.1–13.8 × 1.5–2.9 μm vs. 11.5–19.34 × 2.8–5.4 µm vs. 4.6–9.1 × 1.7–4.3 μm). The conidiogenous cells of *M. chinense* (HMAS 354069, holotype) are longer than those of *M. hainanense* (HMAS 352156, holotype) and *M. brevisporum* (HMAS 354068, holotype) (4.2–22.9 × 1.7–2.3 μm vs. 16.3–22.4 × 4.1–5.7 µm vs. 1.4–9.6 × 0.9–3.0 μm) (Liu et al. 2022). In addition, the colonies of *M. chinense* (HMAS 354069, holotype) are predominantly white, while those of *M. brevisporum* (HMAS 354068, holotype) appear white overall and grow from the center in a concentric ring-like pattern. Therefore, based on morphology and phylogeny, this fungus is established as *Microdochium chinense* sp. nov.

#### Microdochium wuzhishanense

Taxon classificationFungiAmphisphaerialesAmphisphaeriaceae

Y.X. Shang, X.G. Yan, C.C. Ai & X.G. Zhang sp. nov.

56B96150-95E4-5905-9959-C9C8DF7149F3

862174

Fig. 5

##### Type.

China • Hainan Province: Wuzhishan National Nature Reserve, on diseased leaves of *Symplocos sumuntia* Buch-Ham. ex D.Don, 2 December 2024, X.G.Yan (HMAS 354377, holotype), ex-type living culture SAUCC14246-4 = SAUCC14246-4b.

**Figure 5.**
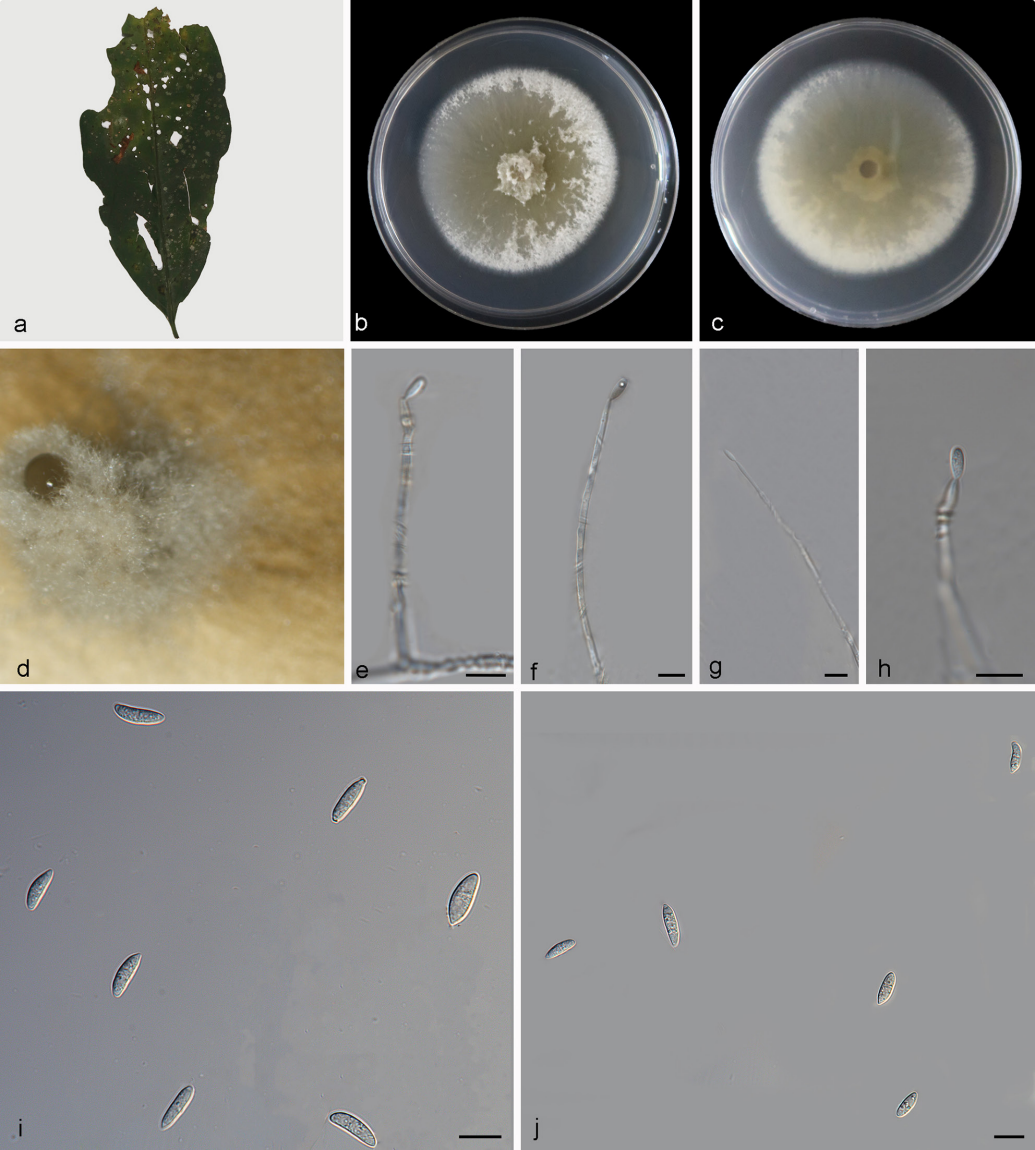
*Microdochium wuzhishanense* (SAUCC14246-4, ex-type). **a**. A leaf of the host plant *Symplocos sumuntia*; **b, c**. Colony on PDA from above and below after 7 days; **d**. Colony overview with conidiomata; **e–h**. Conidiogenous cells with conidia; **i, j**. Conidia. Scale bars: 10 μm (**e–j**).

##### Etymology.

Refers to the collecting site of Wuzhishan National Nature Reserve in Hainan Province, China.

##### Description.

Associated with diseased leaves of *Symplocos sumuntia*. **Asexual morph on PDA: *Mycelia*** superficial, membranous and immersed, hyaline, branched, septate, smooth, 1.7–2.8 μm wide (x̄ = 2.19 μm, *n* = 25). ***Conidiophores*** arising from hyphae, inconspicuous, often reduced to conidiogenous cells. ***Conidiogenous cells*** monoblastic, terminal, solitary, cylindrical, occasionally curved or swollen, hyaline, smooth-walled, 6.8–17.7 × 1.7–3.1 μm (x̄ = 10.3 × 2.4 μm, *n* = 15). ***Conidia*** solitary, hyaline, smooth-walled, elliptical to fusiform or subpisiform, straight or slightly curved, 0–3-septate, 8.5–14.6 × 2.4–4.3 μm (x̄ = 11.3 × 3.6 μm, *n* = 40). ***Chlamydospores*** not observed. **Sexual morph**: Undetermined.

##### Culture characteristics.

Cultures were incubated on PDA at 25 °C in the dark. Colonies attained 61–64 mm in diameter after 7 days, with a daily growth rate of 8.7–9.1 mm. Colonies subcircular; surface tawny-yellow, reverse pale yellowish-green. Mycelium abundant at the colony center and margin, but sparse or locally absent in intermediate areas. Sporulation commenced on PDA after 14 days of incubation, with conidia produced in slimy masses.

##### Notes.

Based on phylogenetic analysis combined with four sequences (ITS, LSU, *RPB2*, and *TUB2*), *Microdochium wuzhishanense* (SAUCC14246-4 and SAUCC14246-4b) constitutes an independent clade, closely related to *M. bambusae* (SAUCC1866-1 and SAUCC1862-1) and *M. bambusarum* (SAUCC7611-3 and SAUCC6699-4). *Microdochium wuzhishanense* (SAUCC14246-4, ex-type) is distinguished from *M. bambusae* (SAUCC1862-1, ex-type) by 25/558, 34/872, 15/875, and 56/467 characters in ITS, LSU, *RPB2*, and *TUB2* sequences, respectively, excluding gap sites. *Microdochium wuzhishanense* (SAUCC14246-4, ex-type) is distinguished from *M. bambusarum* (SAUCC7611-3, ex-type) by 3/536, 9/869, 78/898, and 13/720 characters in ITS, LSU, *RPB2*, and *TUB2* sequences, respectively, excluding gap sites. Morphologically, the conidia of *M. wuzhishanense* (HMAS 354377, holotype) are shorter than those of *M. bambusae* (HMAS 352651, holotype) Jie Zhang, Zhao X. Zhang & Z. Li and *M. bambusarum* (HSAUP 7611-3, holotype) Y.X. Shang, Z. Li bis & X.G. Zhang (8.5–14.6 × 2.4–4.3 μm vs. 13.0–17.0 × 2.5–3.5 μm vs. 11.6–14.8 × 3.5–4.5 μm). The conidiogenous cells of *M. wuzhishanense* (HMAS 354377, holotype) are shorter than those of *M. bambusae* (HMAS 352651, holotype) and longer than those of *M. bambusarum* (HSAUP 7611-3, holotype) (6.8–17.7 × 1.7–3.1 μm vs. 17.4–30.0 × 2.5–3.0 μm vs. 6.3–7.0 × 2.0–2.8 µm) (Zhang et al. 2023; Shang et al. 2025). In addition, the colonies of *M. wuzhishanense* (HMAS 354377, holotype) are tawny-yellow, while colonies of *M. bambusarum* (HSAUP 7611-3, holotype) and *M. bambusae* (HMAS 352651, holotype) are gray-white with regular margins. Therefore, based on morphology and phylogeny, this fungus is established as *Microdochium wuzhishanense* sp. nov.

## Discussion

Prior morphological confusion with morphologically similar genera, such as *Fusarium*, has long hindered accurate species identification, whereas the integration of multilocus molecular data has greatly improved the taxonomic delimitation of this genus. The four-gene combination (ITS, LSU, *RPB2*, and *TUB2*) has become the most reliable molecular marker system for distinguishing closely related *Microdochium* species, following the systematic revision of Hernández-Restrepo et al. (2016). Consistent with this standardized molecular framework, the present study utilized the same four genetic loci to clarify the taxonomic status of eight fungal strains collected from tropical host plants in Hainan Province. Both ML and BI phylogenetic analyses yielded stable topologies. Notably, all four newly established lineages received maximum statistical support (MLBS/BYPP = 100/1), unambiguously separating the examined isolates into four independent monophyletic lineages. Combined with stable and distinguishable morphological characteristics of conidia and conidiogenous cells, these well-delimited lineages were formally defined as four novel species: *Microdochium acronychicola*, *M. brevisporum*, *M. chinense*, and *M. wuzhishanense*.

Phylogenetically, the four newly described species exhibited clear interspecific divergence within the genus. Among them, *M. wuzhishanense* showed close phylogenetic affinity with two bamboo-inhabiting species, *M. bambusae* and *M. bambusarum*, indicating conserved host adaptation to bamboo plants. In addition, *M. chinense* (SAUCC1516001 and SAUCC1516002) and *M. brevisporum* (SAUCC785206 and SAUCC785207) formed a strongly supported sister clade (MLBS/BYPP = 90/1), which further clustered together with *M. hainanense* (SAUCC210781 and SAUCC210782) with high branch support values (MLBS/BYPP = 99/1). The clade comprising the above three species was further grouped with *M. acronychicola* (SAUCC620701 and SAUCC620702) to form a large composite lineage; although this major node displayed a relatively low ML bootstrap value (<70), it received a credible Bayesian posterior probability (BYPP = 0.94), which sufficiently validated its phylogenetic stability. Such topological incongruence between ML and BI analyses is commonly observed in recently diverged fungal lineages. The low bootstrap support is presumably due to limited informative nucleotide sites, low variation in the conserved LSU region, and genetic heterogeneity among different gene fragments, reflecting recent rapid speciation events within this *Microdochium* lineage. Despite minor differences in phylogenetic support, the consistent morphological diagnostic traits in conidial shape, septation, and conidiogenous cell structure further validated the taxonomic independence of the four new species.

Based on publicly accessible biodiversity data from the Global Biodiversity Information Facility (GBIF), *Microdochium* exhibits a global distribution pattern, with abundant records from temperate zones and gradually increasing documentation from tropical and subtropical regions (Crous et al. 2018, 2019; Liang et al. 2019; Liu et al. 2022). Hainan Province, with a typical tropical humid climate, is an important distribution area for *Microdochium*. The four new species discovered in this study effectively supplement the tropical species diversity of *Microdochium*, enrich the global species catalog, and further improve the research status of this genus in South China. The results confirm that tropical forest ecosystems harbor abundant potential *Microdochium* resources.

The host range of *Microdochium* is broader than previously recognized. Although *Poaceae* constitutes the dominant host family, accumulating evidence demonstrates that this genus can colonize phylogenetically diverse plant hosts (Liu et al. 2022; Zhang et al. 2023; Shang et al. 2025). In this study, the newly described species were isolated from three distinct host plants, including an unidentified bamboo species, *Acronychia pedunculata* (*Rutaceae*), and *Symplocos sumuntia* (*Symplocaceae*). Notably, *Acronychia pedunculata* and *Symplocos sumuntia* are reported here as two novel host families for *Microdochium*. This host expansion indicates that members of *Microdochium* possess stronger host adaptability than previously assumed, and the non-graminaceous woody hosts may provide unique microhabitats for cryptic *Microdochium* species. These findings improve understanding of the ecological plasticity and host spectrum within this genus.

Members of *Microdochium* are commonly reported as plant pathogens, mainly infecting grasses and causing leaf blight and snow mold diseases (Crous et al. 2019; Liang et al. 2019). Some species can also live as endophytes or saprobes in plant tissues (Mandyam et al. 2010; Shen et al. 2014). Previous studies have proven that these fungi can change their trophic modes under different host and environmental conditions (Yang et al. 2023). In the present work, all fungal strains were isolated from diseased leaves of *Bambusoideae* sp., *Acronychia pedunculata*, and *Symplocos sumuntia* in Hainan Province. However, artificial inoculation tests were not performed to confirm their pathogenicity. For this reason, it remains unclear whether these fungi act as true pathogens, opportunistic strains, or latent endophytes in host plants. Further investigations, such as pathogenicity tests and field surveys, are necessary to better understand their ecological roles in tropical environments. Overall, the findings enrich the species diversity, host information, and phylogenetic data of *Microdochium*, which will support further ecological and pathological research on this fungal genus.

## Conclusion

Samples of diseased and decaying leaves were collected from Hainan Province, China. Multiple strains of the genus *Microdochium* were isolated using single-spore and tissue isolation techniques. Four new species of *Microdochium*: *M. acronychicola*, *M. brevisporum*, *M. chinense*, and *M. wuzhishanense* were identified based on morphological characteristics and multilocus phylogenetic analysis. Additional novel and newly recorded species of *Microdochium* are likely to be isolated from diverse host plants worldwide, further enhancing the known species diversity of this genus.

## Supplementary Material

XML Treatment for Microdochium acronychicola

XML Treatment for Microdochium brevisporum

XML Treatment for Microdochium chinense

XML Treatment for Microdochium wuzhishanense

## Additional information

### Artificial Intelligence (AI) use

The authors accept full responsibility for the content of the manuscript, including the disclosure of any use of AI.

No AI tools were used in the preparation of this manuscript.

### Author ORCIDs

Y.-J. Wang https://orcid.org/0000-0002-9775-3716

X.-G. Yan https://orcid.org/0009-0005-6257-3490

S. Wang https://orcid.org/0000-0002-7376-7638

Y. Jiang https://orcid.org/0009-0003-1292-610X

Y.-X. Shang https://orcid.org/0009-0000-7211-5139

W.-W. Liu https://orcid.org/0009-0009-3477-194X

X.-S. Wang https://orcid.org/0009-0005-1127-7368

J.-Y. Sheng https://orcid.org/0009-0003-3882-6748

X.-G. Zhang https://orcid.org/0000-0001-9733-8494

C.-C. Ai https://orcid.org/0009-0002-3934-7944

### Data availability

All of the data that support the findings of this study are available in the main text.
